# Photocatalytic degradation of rhodamine B using zinc oxide/silver nanowire nanocomposite films under ultraviolet irradiation

**DOI:** 10.1098/rsos.241967

**Published:** 2025-06-18

**Authors:** Noah Jang, June Soo Kim, Hyunjun Kim, Da Ye Kim, Yujin Nam, Maeum Han, Seong Ho Kong

**Affiliations:** ^1^ Kyungpook National University, Daegu, Republic of Korea

**Keywords:** photocatalytic degradation, rhodamine B, nanocomposite film, ZnO/Ag nanowire

## Abstract

Water pollution from industrial and household waste presents significant environmental challenges, particularly owing to the widespread use and toxicity of organic dyes such as rhodamine B (RhB). This study investigates the photocatalytic degradation of RhB using composite films composed of zinc oxide (ZnO) and silver nanowires (AgNWs) under ultraviolet (UV) irradiation. ZnO is well known for its strong photocatalytic activity because of its high charge-carrier mobility and ability to generate reactive oxygen species (ROS). However, its relatively large bandgap (approx. 3.3 eV) limits its light absorption primarily to the UV range, restricting its photocatalytic efficiency under visible light. The incorporation of AgNWs is expected to enhance charge separation, increase electron mobility and introduce localized surface plasmon resonance effects, which contribute to improved light absorption and photocatalytic performance. The ZnO/AgNW composite films were synthesized using a sol–gel method and characterized through scanning electron microscopy and energy-dispersive X-ray spectroscopy to analyse the morphology and elemental composition, X-ray diffraction to confirm the crystallinity structure, and UV–visible spectroscopy to determine optical properties and bandgap energy. The bandgap reduction observed in ZnO/AgNW composites, as confirmed by Tauc plot analysis, is attributed to structural modifications, oxygen vacancy formation and plasmonic interactions that enhance charge transfer and light absorption. This enhanced optical response directly contributed to the superior photocatalytic efficiency of the composite. The reduction in bandgap directly influenced the photocatalytic performance of the ZnO/AgNW composites. A lower bandgap extends light absorption into the visible range, allowing the material to use a broader spectrum of incident light. Furthermore, the enhanced charge-carrier separation and increased ROS generation contributed to superior photocatalytic efficiency. As a result, the ZnO/AgNW composite films achieved a 90% degradation efficiency of RhB within 40 min of UV exposure, demonstrating a significant improvement over conventional ZnO-based photocatalysts. These findings highlight the potential of ZnO/AgNW nanocomposites as efficient, reusable and scalable solutions for water purification and environmental remediation applications.

## 1. Introduction

Rapid industrialization and urbanization efforts in recent times have led to severe water pollution problems globally, with organic dyes being one of the primary pollutants found in wastewater [1,2]. These dyes are extensively used in textile, paper, food and cosmetics industries and pose significant environmental and health hazards owing to their toxicity and persistence in the environment [3]. Rhodamine B (RhB) is a commonly used dye that adversely affects aquatic ecosystems and causes severe health issues in humans [4]. These issues clearly highlight the urgent need to develop efficient and sustainable methods for removing pollutants from aquatic environments [5].

Environmental regulations aimed at mitigating the impacts of hazardous chemicals on ecosystems and human health have become increasingly stringent [6]. For instance, the European Union’s Water Framework Directive mandates the reduction of priority substances, including specific dyes, to near-zero concentrations. Similarly, the United States Environmental Protection Agency enforces regulations that limit the discharge of toxic chemicals into water bodies. Despite existing regulations, conventional water treatment methods relying on adsorption, coagulation and biological processes are often inadequate for effectively removing complex dye pollutants. This emphasizes the need for advanced materials and innovative approaches in water treatment technologies [7,8].

Photocatalysis using semiconductor materials has emerged as a promising solution for the degradation of organic pollutants [9,10]. Zinc oxide (ZnO) is particularly favoured for this purpose because of its high photosensitivity, chemical stability and nontoxicity [11,12]. The ability of ZnO to generate electron–hole pairs under ultraviolet (UV) irradiation makes it an effective photocatalyst [13,14]. However, ZnO exhibits high electron–hole recombination rates, significantly reducing its photocatalytic efficiency. This rapid recombination prevents efficient charge transfer, limiting the generation of reactive oxygen species (ROS), which are crucial for pollutant degradation [15,16]. Additionally, ZnO has a relatively large bandgap (approx. 3.37 eV), restricting its visible light absorption and making it inefficient under solar irradiation. To overcome these limitations, various strategies have been explored, including the incorporation of noble metals such as gold (Au), platinum (Pt), palladium (Pd) and silver (Ag) to enhance the photocatalytic performance of ZnO. [17–19].

Among these options, silver nanowires (AgNWs) have shown exceptional potential owing to their unique one-dimensional structures that facilitate better charge transport and provide more efficient pathways for electron conduction compared to nanoparticles [19]. AgNWs function as electron acceptors and conductive pathways, effectively separating photogenerated charge carriers. This suppression of electron–hole recombination significantly enhances photocatalytic activity by prolonging charge-carrier lifetimes. Additionally, AgNWs contribute to localized surface plasmon resonance (LSPR), which improves light absorption and broadens the spectral response of ZnO into the visible range [20]. The greater active surface area of the AgNWs offers more sites for the generation of ROS, significantly boosting the overall photocatalytic efficiency [21,22]. The uniform distribution of ZnO on the AgNWs enhances light absorption and increases the number of active sites available for the degradation of organic pollutants, such as RhB [23,24].

While previous studies have investigated TiO_2_-based and Cu_2_O-based photocatalysts, their efficiency has been limited by inefficient charge separation or narrow spectral absorption. ZnO, despite its advantages, still suffers from rapid electron–hole recombination and limited visible light absorption. Unlike nanoparticle-based silver dopants, AgNWs provide a continuous conductive network that facilitates efficient charge transfer, reducing energy loss and enhancing photocatalytic efficiency. Furthermore, most previous work on ZnO/AgNW composites have focused primarily on fundamental characterization, lacking real-world application assessments. Additionally, there is still limited understanding of the detailed charge transfer mechanisms between ZnO and AgNWs, necessitating further investigation.

**Figure 1.**
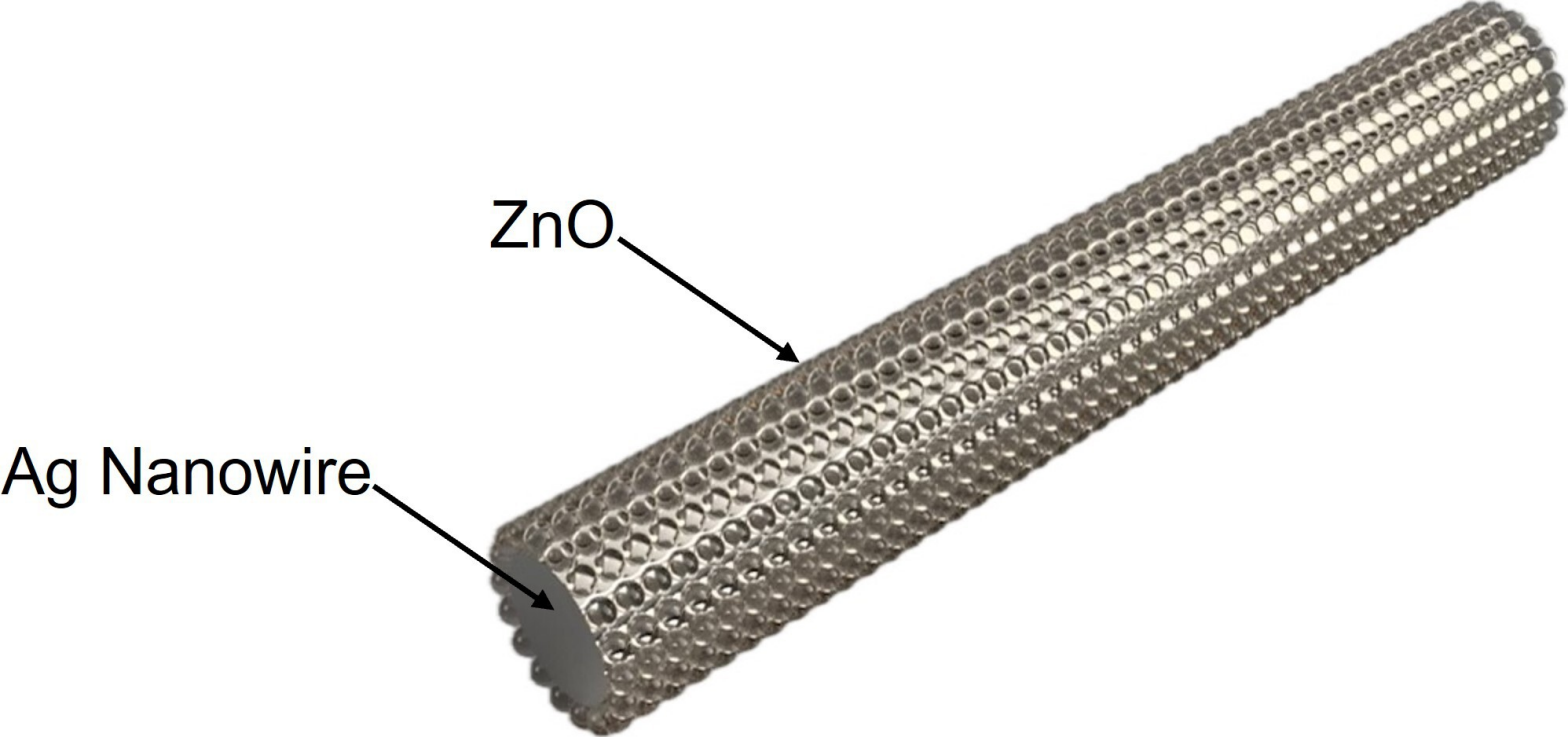
Schematic illustration of the ZnO/AgNW for photocatalytic application.

In this study, we investigate the effect of AgNW incorporation on the optical and electronic properties of ZnO. AgNWs are expected to enhance charge separation, introduce LSPR and improve light absorption efficiency, ultimately reducing the bandgap of ZnO. Figure 1 presents a schematic representation of the ZnO/AgNW composite, where ZnO is uniformly coated onto the surface of AgNWs, forming a hybrid nanostructure. This architecture facilitates efficient charge transfer and optimizes light absorption, thereby enhancing photocatalytic activity. The bandgap modification and its impact on photocatalytic performance were analysed in detail using optical characterization techniques.

This reduction in bandgap resulted in broadened light absorption into the visible range, allowing for more efficient use of incident photons. Additionally, the improved charge separation and enhanced electron mobility facilitated by AgNWs suppressed electron–hole recombination, further increasing photocatalytic efficiency. The photocatalytic performance of the ZnO/AgNW composite was evaluated through the degradation of RhB under UV irradiation, showing a substantial enhancement compared to pure ZnO.

The outstanding photocatalytic efficiencies of the ZnO/AgNW composites in this study can be attributed to multiple synergistic effects, including reduced bandgap energy, enhanced charge transfer and increased ROS generation via LSPR. These improvements enable more efficient degradation of organic pollutants, highlighting ZnO/AgNW composites as promising candidates for sustainable environmental remediation and water purification applications.

## 2. Experimental procedures

### 2.1. Materials

Zinc acetate dihydrate (Zn(CH_3_COO)_2_·2H_2_O), 2-methoxyethanol (CH_3_OCH_2_CH_2_OH) and acetone ((CH_3_)_2_CO) were purchased from Sigma-Aldrich. The AgNWs were obtained from Sigma-Aldrich and had specific characteristics, including a molar mass of 107.87 g mol^−1^, flash point of 12°C (closed cup) and density of 0.785 g ml^−1^ at 25°C; further, the AgNWs used in this study had a diameter of 60 nm and length of 10 μm, with a concentration of 0.5% (in an isopropyl alcohol (IPA) suspension).

### 2.2. Synthesis of the hybrid zinc oxide/silver nanowire nanocomposite

A ZnO seed layer was first synthesized using the sol–gel method. Initially, a 0.3 M solution of zinc acetate dihydrate was prepared by dissolving 0.66 g of the compound in 10 ml of 2-methoxyethanol [25,26]. This solution was heated to 70°C and stirred continuously for 1 h to ensure complete dissolution and promote the hydrolysis and polycondensation reactions characteristic of the sol–gel process. The ZnO precursor solution was then filtered using a 0.2 µm syringe filter to remove the undissolved particles.

Glass substrates were cleaned using a sequential washing process involving acetone, IPA and deionized water to remove any residual contaminants. Next, the substrates were subjected to UV irradiation for 10 min to enhance surface cleanliness.

The purified ZnO precursor solution was mixed with an equal volume (3 ml) of the AgNW suspension (AgNWs dispersed in IPA, 0.5% by weight) to ensure uniform dispersion. This mixture was then spin-coated onto the UV-irradiated glass substrates at 2500 r.p.m. for 30 s. The coated substrates were oxidized at 250°C, and this process was repeated for 10 cycles to achieve a uniform film. Finally, the samples were annealed at 600°C for 1 h in air to enhance crystallinity and adhesion. The heating rate was set to 5°C min^−1^.

The optimized fabrication method enables the formation of a well-integrated ZnO/AgNWs composite, leveraging the synergistic properties of both materials. By ensuring uniform film deposition and controlled crystallization, this approach enhances charge separation, increases surface area and improves the stability of the hybrid nanocomposite. This method provides a reproducible and scalable pathway for developing ZnO/AgNWs hybrids suitable for photocatalytic applications.

### 2.3. Characterization

The structural properties of the ZnO/AgNWs nanocomposites, including thickness, grain size and elemental composition, were characterized by field-emission scanning electron microscopy (FE-SEM). The crystalline structure of ZnO was evaluated using X-ray diffraction (XRD). Energy-dispersive X-ray spectroscopy (EDS) was used to determine the elemental composition of the prepared samples. Raman spectroscopy was used to identify the vibrational modes and confirm the successful incorporation of Ag into the ZnO matrix, which is crucial for understanding the enhanced photocatalytic activity. The optical properties of the samples were examined using UV–visible (UV–Vis) spectrophotometry, and the bandgap energy was estimated using the Tauc plot method by analysing the absorption characteristics. Electrical measurements were conducted using a Keithley 4200 semiconductor parameter analyser to assess the electronic properties of the nanocomposites.

## 3. Results and discussion

### 3.1. Morphology of the zinc oxide/silver nanowire nanocomposite

**Figure 2.**
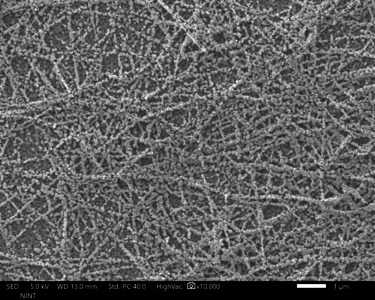
Top-view FM-SEM image of the ZnO/AgNWs.

The structural properties and elemental composition of the ZnO/AgNW nanocomposite were extensively characterized using FE-SEM and EDS. The FE-SEM image shown in figure 2 illustrates that the ZnO nanoparticles are uniformly seeded along the AgNWs, forming well-aligned structures with a high degree of uniformity and dispersion. This well-ordered alignment of the ZnO particles is critical because it enhances the photocatalytic effect by increasing the surface area available for light absorption and catalytic reactions. The densely packed arrays of ZnO particles that are smooth and faceted suggest high crystallinity, which is beneficial for photocatalysis as it reduces the number of defects that could act as electron–hole recombination centres.

**Figure 3.**
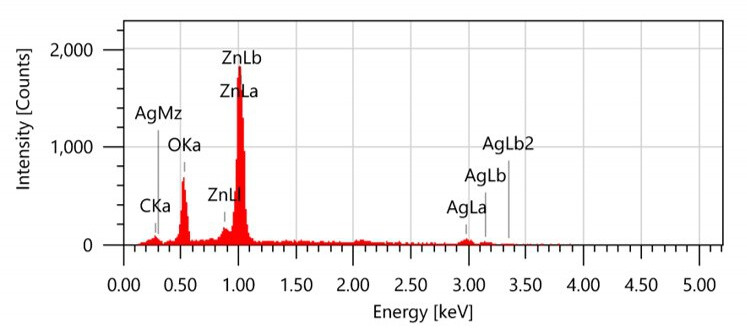
EDS analysis of the ZnO/AgNWs for elemental characterization.

The integration of AgNWs in the ZnO matrix, as seen in the FE-SEM image, results in a one-dimensional structure that further enhances photocatalytic efficiency. This is achieved by improving electron mobility and facilitating better charge separation, both of which are essential for reducing the recombination rate and increasing ROS generation. The EDS analysis results shown in figure 3 confirm the presence of both Zn and Ag, which are distributed uniformly across the nanowire structures. This uniform distribution is crucial because it ensures consistent photocatalytic activity across the composite material.

The well-ordered alignment of ZnO nanoparticles along the AgNWs significantly enhances the photocatalytic performance by increasing light absorption, optimizing charge transport and suppressing electron–hole recombination. This structured composite provides a greater number of active sites, facilitating efficient interactions between the photocatalyst and target pollutants. Additionally, the enhanced surface area from the nanowire morphology, combined with the plasmonic properties of AgNWs, promotes the efficient generation of ROS, which are crucial for the effective degradation of organic pollutants such as RhB.

### 3.2. Structural analysis of the zinc oxide/silver nanowire nanocomposite

**Figure 4.**
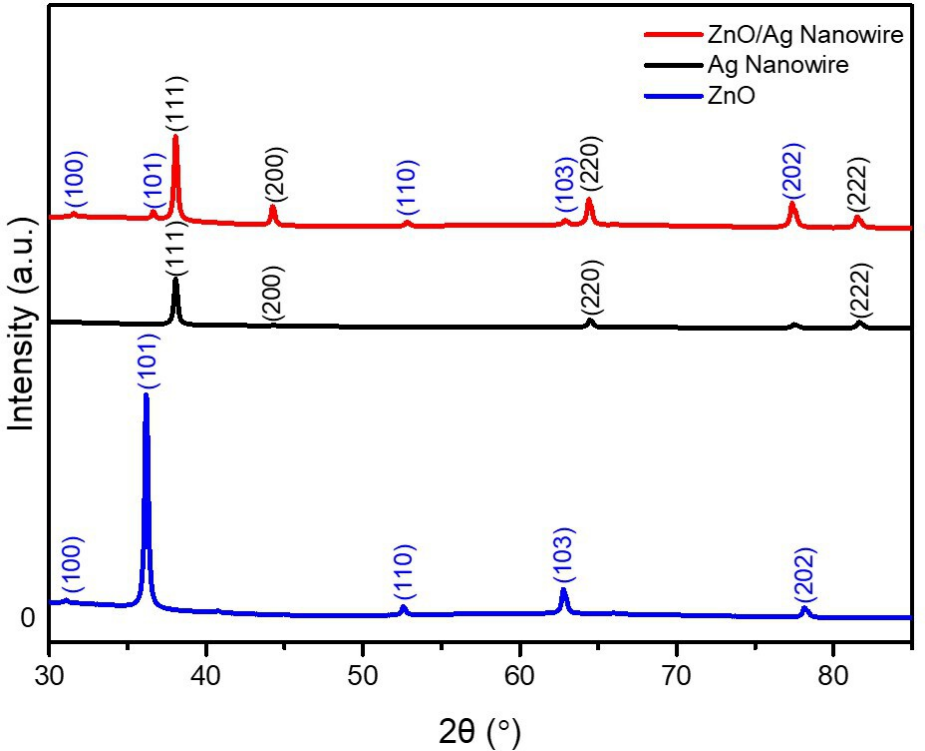
XRD analysis for structural characterizations of ZnO, AgNWs and ZnO/AgNW composite.

The crystalline structure and quality of the ZnO/AgNW nanocomposite were analysed using XRD to confirm phase purity, crystallinity and structural integrity, as shown in figure 4. The XRD pattern confirms the crystalline nature of the nanocomposite, revealing distinct peaks corresponding to the ZnO wurtzite structure and metallic Ag. The diffraction peaks at (100), (101), (110), (103) and (202) planes are characteristic of the hexagonal wurtzite structure of ZnO, confirming its high crystallinity and phase purity. The observed peaks at the (100), (101), (110), (103) and (202) planes of ZnO as well as the (111), (200), (220) and (222) planes of the AgNWs validate the successful synthesis of the composite. Meanwhile, the (111), (200), (220) and (222) diffraction peaks correspond to the face-centred cubic (FCC) structure of Ag, indicating that the metallic AgNWs are well-preserved without oxidation. The coexistence of these well-defined structures suggests that AgNWs are effectively integrated with ZnO without disrupting its intrinsic crystallinity [27,28].

The wurtzite structure of ZnO is characterized by high electron mobility and a wide bandgap, which play significant roles in enhancing the photocatalytic activity by facilitating efficient charge separation and reducing the recombination rate [29,30]. This structure provides a built-in electric field that enhances charge separation, while the high crystallinity ensures minimal defect states that could act as recombination centres. The presence of AgNWs further strengthens this effect by acting as an efficient electron transport pathway, reducing energy loss and suppressing electron–hole recombination. This is particularly advantageous in photocatalysis, where maintaining prolonged charge separation is key to effective pollutant degradation. The synergistic effect between ZnO and AgNWs optimizes charge transport, enabling faster electron migration and improving photocatalytic efficiency.

**Figure 5.**
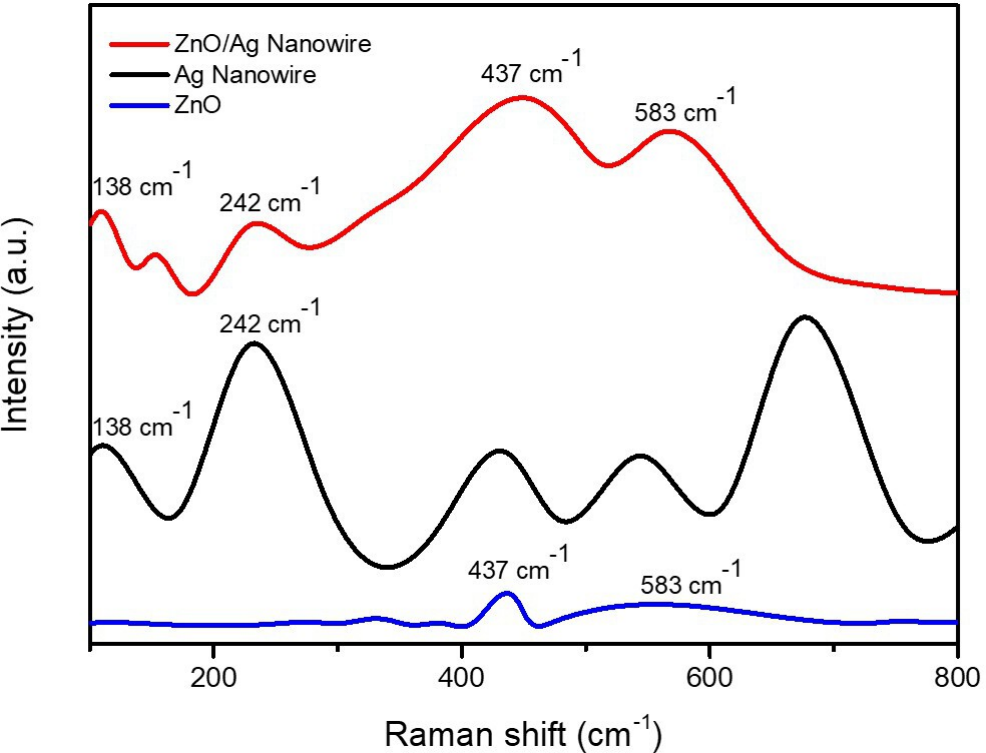
Raman spectra of ZnO, AgNWs and ZnO/AgNW composite highlighting the characteristic peaks.

Raman spectroscopy was employed to identify the vibrational modes and confirm the successful incorporation of Ag into the ZnO matrix, as shown in figure 5. The Raman spectrum of the ZnO/AgNW composite exhibited significant peaks at 233, 440 and 568 cm^−1^ corresponding to the AgNW, as well as at 432 and 538 cm^−1^ corresponding to the ZnO structure. The peaks at 432 and 538 cm^−^¹ confirm the characteristic vibrational modes of ZnO, while the additional peaks at 233, 440 and 568 cm⁻¹ indicate the presence of AgNWs. The intensity and shift of these peaks suggest a strong interfacial interaction between ZnO and Ag, which enhances charge transfer dynamics [31–35].

The distinct peaks of both ZnO and Ag observed in the XRD patterns confirm the successful integration of both materials without compromising their individual crystal structures. This preservation of individual structures while maintaining strong electronic coupling is crucial for maximizing the photocatalytic performance of the composite. This coexistence allows the composite to benefit from the photocatalytic properties of both materials. The ZnO wurtzite phase contributes to efficient charge separation through its polar surfaces, while AgNWs act as electron mediators, preventing recombination losses and enhancing reaction kinetics. Specifically, the well-defined wurtzite structure of ZnO enhances the photocatalytic activity by creating internal electric fields that drive the separation of the photogenerated electron–hole pairs, while the FCC structure of Ag, indicated by its (111), (200), (220) and (222) peaks, enhances conductivity and electron mobility. The FCC structure of Ag provides a high density of free electrons, facilitating rapid electron transport and reducing resistive losses in the composite. Additionally, LSPR from Ag enhances light absorption, extending the photocatalytic response into the visible range.

The high crystallinity of the ZnO/AgNW composite, as indicated by the sharp XRD peaks and confirmed by Raman spectroscopy, is essential for improving the photocatalytic performance. Higher crystallinity reduces defect density, preventing charge trapping and ensuring effective electron–hole pair utilization in catalytic reactions. The presence of the AgNWs not only enhances electron mobility but also generates ROS, which are vital for the effective breakdown of organic pollutants such as RhB. The combination of these properties renders the ZnO/AgNW composite highly effective for environmental remediation applications, particularly in scenarios in which long-term stability and high efficiency are required. These combined structural and electronic enhancements positions the ZnO/AgNW composite as a highly efficient photocatalyst for environmental remediation, providing long-term stability, improved charge use and enhanced ROS generation.

### 3.3. Optical properties of the zinc oxide/silver nanowire nanocomposite

To comprehensively evaluate the effects of incorporating AgNWs into ZnO, a comparative analysis of the optical properties of pure ZnO and ZnO/AgNWs was conducted. The AgNWs were selected as the benchmark owing to their one-dimensional structures, which inherently enhance the photocatalytic properties compared to bulk ZnO. By comparing these results with those of the ZnO/AgNW composite, the additional advantages provided by the AgNWs, such as improved electron mobility, enhanced charge separation and increased ROS generation, can be better quantified. This approach allows isolation and clear understanding of the synergistic interactions that contribute to the superior photocatalytic performance of the ZnO/AgNW composite.

**Figure 6.**
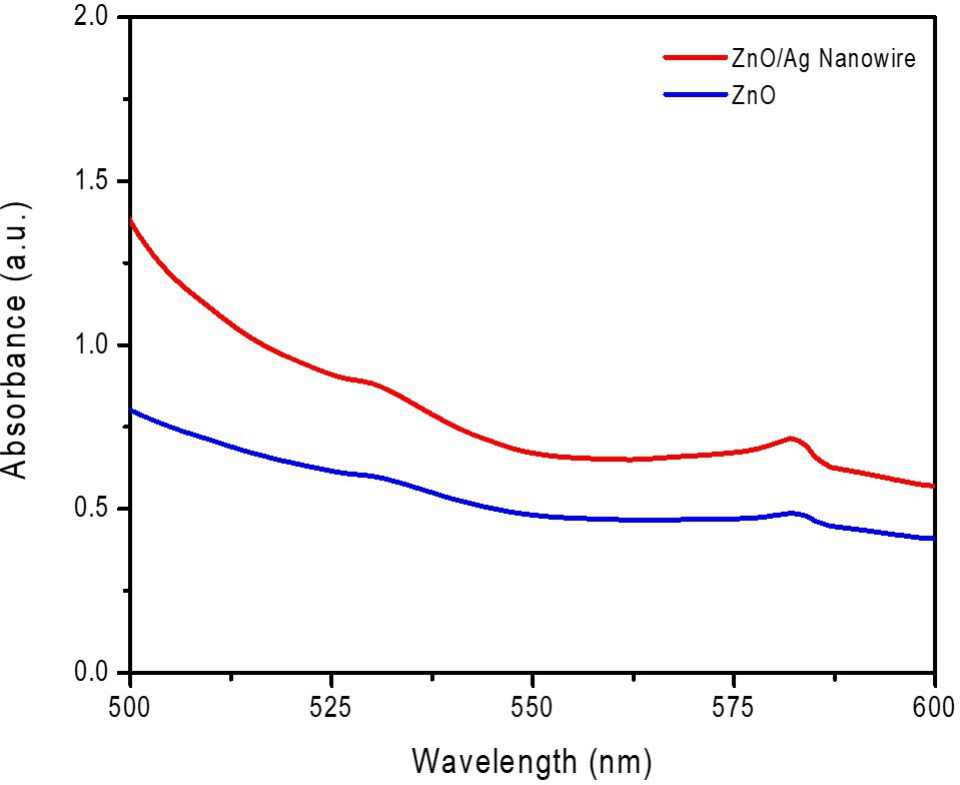
UV–Vis absorption spectra of ZnO and ZnO/AgNW composite in the wavelength range of 500−600 nm.

The optical properties were thoroughly investigated using UV–Vis spectrophotometry, and these results are shown in figure 6. AgNWs were specifically chosen for this study because of their unique ability to enhance optical and electronic properties of ZnO. Unlike conventional noble metal nanoparticles, AgNWs provide a continuous conductive pathway for charge transport, thereby improving electron mobility and suppressing electron–hole recombination. The incorporation of AgNWs also induces an LSPR effect, which enhances light absorption in the visible range by generating strong localized electromagnetic fields. This plasmonic enhancement not only increases the photocatalytic efficiency by facilitating charge separation but also enables a broader spectral response, making the composite more effective under both UV and visible light. Furthermore, the one-dimensional morphology of AgNWs provides superior electron conduction paths compared to isolated nanoparticles, reducing energy loss and improving the efficiency of charge transfer within the ZnO matrix.

The bandgap energy ($E_{g}$) was determined using the Tauc plot method, which uses UV–Vis absorption data to establish the relationship between the optical absorption coefficient (α) and the incident photon energy (hv). This approach allows for a precise estimation of the optical bandgap by analysing the absorption characteristics of ZnO and ZnO/AgNWs. For direct bandgap semiconductors such as ZnO, the Tauc equation is given by:


$${\left(\alpha hv\right)}^{2}=A\left(hv-\;E_{g}\right),$$

where A is a material-dependent constant. The absorption coefficient (α) is calculated using the equation:


$$\alpha =\;\frac{2.303A}{d},$$

where A is the absorbance and d is the sample thickness. To determine the bandgap energy, a Tauc plot was constructed by plotting ${\left(\alpha hv\right)}^{2}$ versus hv, and the linear portion of the curve was extrapolated to the energy axis intercept, which corresponds to ${\left(\alpha hv\right)}^{2}=0$, to estimate the optical bandgap. To determine the bandgap energy from the experimental data, the Tauc plot was constructed by fitting the linear region of the ${\left(\alpha hv\right)}^{2}$ versus hv curve and using the linear equation:


$${\left(\alpha hv\right)}^{2}=\alpha \left(hv\right)+b,$$

where α is the slope and b is the intercept. The bandgap energy was obtained using the equation:


$$E_{g}=-\frac{b}{a}.$$

By applying the Tauc plot method to the UV–Vis absorption data, the optical bandgap was determined by extrapolating the linear region of the ${\left(\alpha hv\right)}^{2}$ versus hv plot. The significant bandgap reduction observed in ZnO/AgNW composites (from 2.66 to 1.40 eV) is attributed to LSPR, improved charge-carrier transport and the introduction of additional defect states owing to AgNW incorporation, which collectively enhance photocatalytic performance. The reduction in the bandgap of ZnO from its typical bulk value (approx. 3.3 eV) to 2.66 eV is attributed to specific modifications in the sol–gel synthesis process. In this study, a controlled annealing process at 600°C was employed to induce the formation of oxygen vacancies and defect states within the ZnO lattice. These oxygen vacancies introduce localized energy states near the conduction band (CB), effectively reducing the optical bandgap. Additionally, the repeated spin-coating and oxidation steps ensured a uniform film morphology, which contributed to increased charge trapping and defect formation, further lowering the bandgap.

A lower bandgap energy enhances photocatalytic performance by increasing visible light absorption, thereby improving solar energy use. Additionally, the incorporation of AgNWs facilitates improved charge transport by providing conductive pathways, while the presence of oxygen vacancies reduces electron–hole recombination, ultimately leading to a higher-density ROS generation. A reduced bandgap facilitates easier excitation of electrons from the valence band (VB) to the CB under lower-energy photons, leading to a higher density of photogenerated charge carriers. Furthermore, oxygen vacancies act as charge-trapping centres that prolong charge-carrier lifetimes, effectively reducing recombination rates and increasing photocatalytic efficiency. This improvement results in a greater yield of ROS, which plays a crucial role in the degradation of organic pollutants.

With its enhanced absorption in this spectral range, the ZnO/AgNW composite becomes markedly more efficient at using visible light, leading to notable improvements in its overall photocatalytic efficiency. These findings highlight the suitability of the composite for environmental remediation and other applications that require effective light-driven catalytic processes. Figure 6 presents a comparative analysis of the absorption spectra, highlighting the significantly enhanced light absorption of the ZnO/AgNW composite, particularly in the visible region (500–600 nm). This result strongly supports the role of AgNWs in plasmonic enhancement, confirming their contribution to charge-carrier dynamics and extended spectral response.

### 3.4. Photocatalytic activity of the zinc oxide/silver nanowire nanocomposite

**Figure 7.**
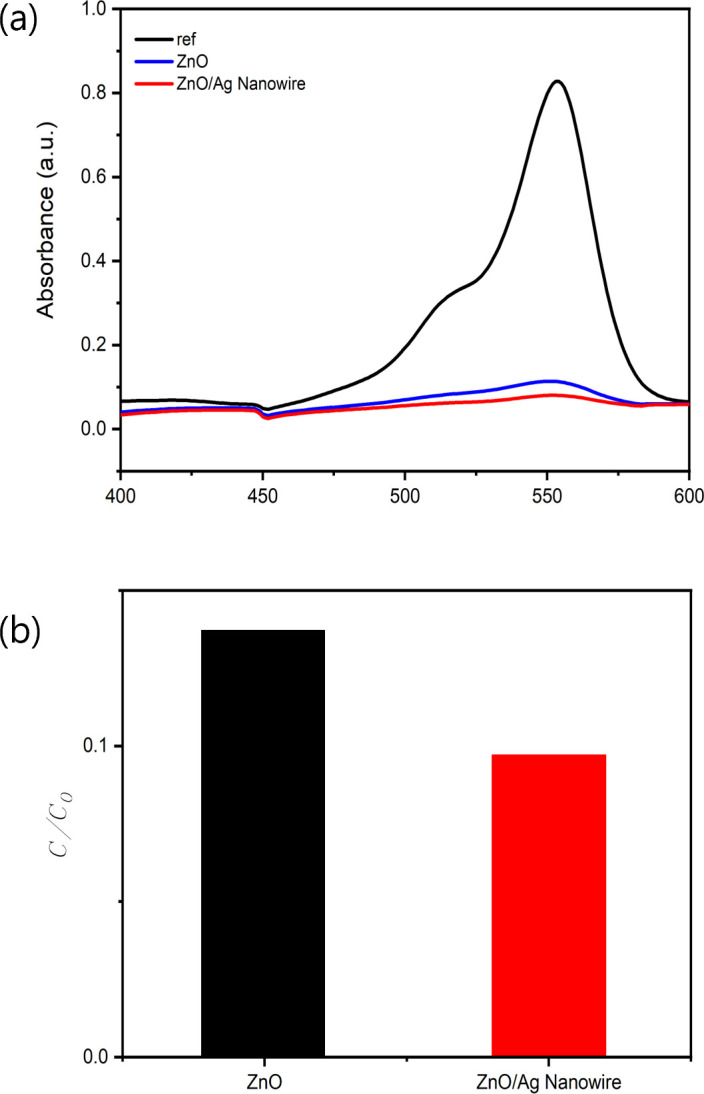
(*a*) UV–Vis absorption spectra of RhB after photocatalysis by ZnO and ZnO/AgNW composite under UV irradiation at 365 nm for 40 min with respect to the reference (initial) value. (*b*) *C*/*C*_0_ values of RhB degradation by ZnO and ZnO/AgNW composite under UV irradiation at 365 nm for 40 min.

As illustrated in figure 7, the photocatalytic activity of the ZnO/AgNW nanocomposite was assessed by monitoring the degradation of RhB dye under UV irradiation at 365 nm for 40 min. The degradation rate of RhB was monitored using UV–Vis absorption spectroscopy, which revealed a rapid decrease in the absorption peak of RhB at 554 nm over time under UV irradiation at 365 nm.

To quantitatively compare the efficiencies of the different photocatalysts, the ratios of the final concentration (*C*) of RhB to its initial concentration (*C*_0_) after 40 min of UV irradiation were calculated. The resulting *C*/*C*_0_ values for the nanocomposites are plotted in a bar graph to visualize their relative performances. The ZnO/AgNW composite exhibited significantly higher photocatalytic efficiency than pure ZnO, achieving the lowest *C*/*C*_0_ value of approximately 0.1 and indicating the most substantial degradation of RhB.

This superior photocatalytic efficiency can be attributed to several key factors. First, the incorporation of AgNWs significantly enhances electron mobility, facilitating more efficient charge separation under UV irradiation. This reduces the recombination rate of the photogenerated electron–hole pairs, thereby prolonging the availability of the charge carriers for the photocatalytic reaction. Additionally, the AgNWs promote the generation of ROS through their surface plasmon resonance (SPR) effect, which further accelerates the degradation process [36]. The synergistic interactions between ZnO and the AgNWs improve the charge separation and increase the number of active sites available for catalytic reactions, leading to the observed enhancement of the degradation of RhB, as reflected by the significantly lower *C*/*C*_0_ value under these specific UV conditions [37].

**Figure 8.**
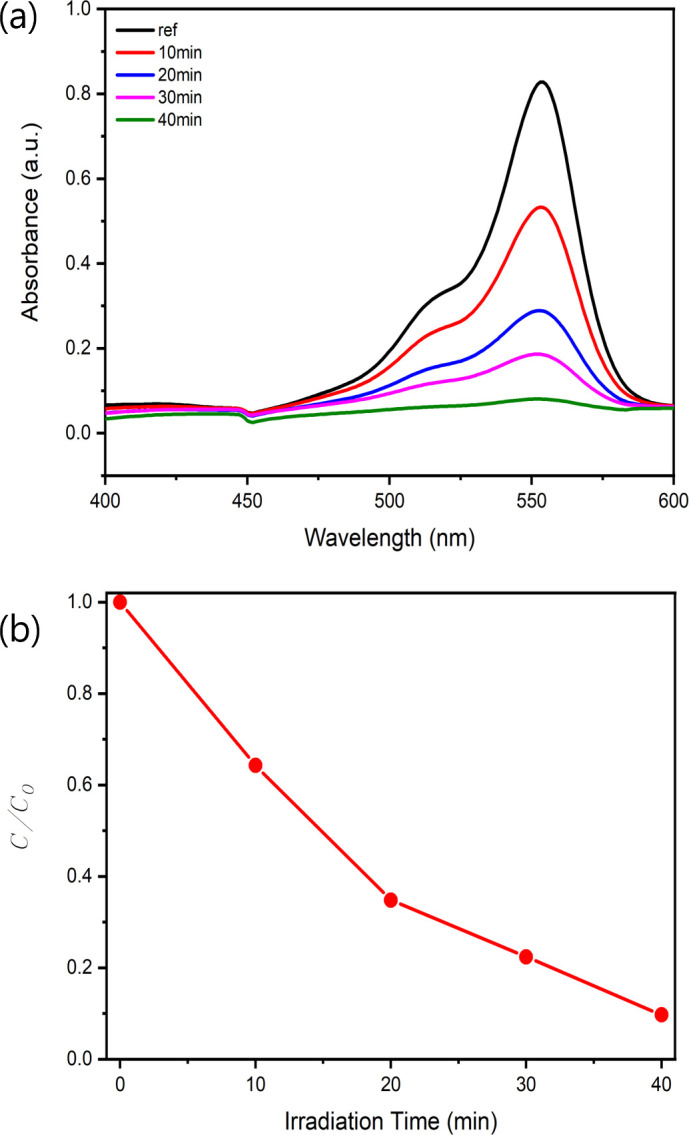
(*a*) UV–Vis absorption spectra of RhB by the ZnO/AgNW composite under UV irradiation at 365 nm for different intervals of time. (*b*) *C*/*C*_0_ values of RhB degradation by the ZnO/AgNW composite under UV irradiation at 365 nm for different intervals of time.

Following the assessment of the photocatalytic activities, the degradation of RhB dye was analysed by monitoring the changes in absorbance within the 400−600 nm wavelength range at intervals of 10, 20, 30 and 40 min under UV irradiation at 365 nm. Figure 8 presents the UV–Vis absorption spectra of RhB at these time points, showing marked decreases in the absorption peak at 554 nm with increasing irradiation time. This decrease in absorbance is directly correlated with the progressive degradation of RhB, highlighting the effectiveness of the ZnO/AgNW nanocomposite as a photocatalyst.

In addition to the absorbance spectra, the RhB degradation efficiency was quantified using the *C*/*C*_0_ ratio, where *C*_0_ is the reference (ref) value at time zero. The resulting *C*/*C*_0_ values were plotted against time, as shown in figure 8, demonstrating a steady decline from 1 to almost 0 as the UV irradiation time increased from 10 to 40 min. The ZnO/AgNW composite exhibited the most pronounced decrease in *C*/*C*_0_, reaching a value close to 0.1, indicating its excellent photocatalytic performance.

To further quantify the photocatalytic efficiency of the ZnO/AgNW composite, we analysed the reaction kinetics using the pseudo-first-order Langmuir–Hinshelwood model, which is commonly used to describe photocatalytic degradation reactions:


$$\ln\left(\frac{C_{0}}{C}\right)=\;kt,$$

where $C_{0}$ and C represent the initial and remaining RhB concentrations at time t, and k is the reaction rate constant.

**Figure 9.**
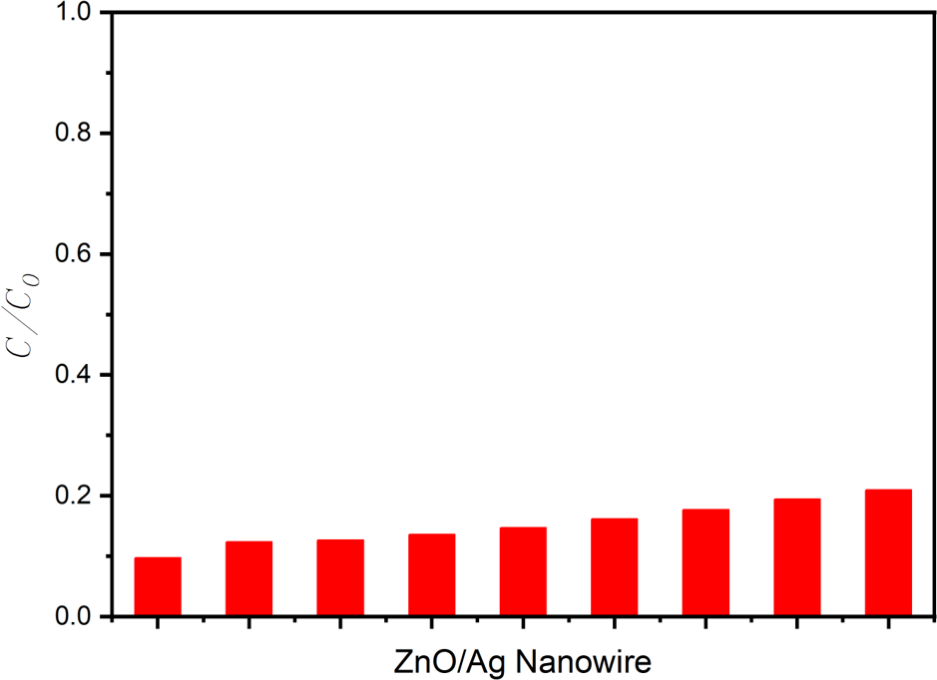
*C*/*C*_0_ values of RhB degradation across repeated photocatalytic cycles using the ZnO/AgNW composite under UV irradiation.

For the ZnO/AgNW system, the rate constant k was determined from the slope of the linear fit in the plot of $ln\left(C_{0}/C\right)$ versus time. The following values were used:

$C_{0}=1$ (normalized RhB concentration at t=0),

C=0.09707 at t=40 min (from figure 9).

Using t=40 min, we obtain


$$k=\;\frac{\ln\left(1.0/0.09707\right)}{40}=0.0054\;{\text{min}}^{-1}.$$

This significantly higher reaction rate compared to conventional ZnO suggests an accelerated degradation process owing to the enhanced charge separation and LSPR effects of AgNWs. The improved kinetics of ZnO/AgNW confirms its superior photocatalytic performance in practical applications.

This analysis clearly illustrates that the ZnO/AgNW nanocomposite not only facilitates a significant reduction in RhB concentration over time but also outperforms pure ZnO and AgNWs alone in terms of photocatalytic efficiency. The sharp declines in both the absorbance and *C*/*C*_0_ values underline the effectiveness of the ZnO/AgNW composite in degrading RhB, driven by the synergistic effects of the composite on the enhanced electron mobility, efficient charge separation and increased generation of ROS through the SPR effect of the AgNWs.

To further evaluate the robustness and reusability of the ZnO/AgNW nanocomposite, the photocatalytic degradation of RhB was evaluated over nine consecutive cycles under identical UV irradiation conditions. Figure 9 shows the *C*/*C*_0_ values recorded for each cycle, starting with an initial *C*/*C*_0_ value of 0.09707 during the first cycle and ending with a *C*/*C*_0_ value of 0.2095 during the ninth cycle. Despite a slight increase in the *C*/*C*_0_ value with each subsequent cycle, the ZnO/AgNW composite maintained a consistently high photocatalytic efficiency throughout the repeated tests.

The high reaction rate constant *k* = 0.0054 min^−1^ obtained from kinetics analysis aligns well with the observed long-term photocatalytic performance in figure 9. Despite multiple degradation cycles, the ZnO/AgNW composite retained a significant portion of its photocatalytic efficiency, confirming its structural stability and sustained high charge transfer efficiency. This reinforces the conclusion that AgNWs effectively enhance both the initial reaction kinetics and long-term reusability, making ZnO/AgNW a highly efficient and durable photocatalyst. The relatively low *C*/*C*_0_ values across all nine cycles highlight the high durability and reusability of the composite, which are essential characteristics for practical environmental remediation applications. This consistent performance underscores the potential of the proposed composite for long-term use in degrading organic pollutants, demonstrating its effectiveness and stability over multiple uses.

**Figure 10.**
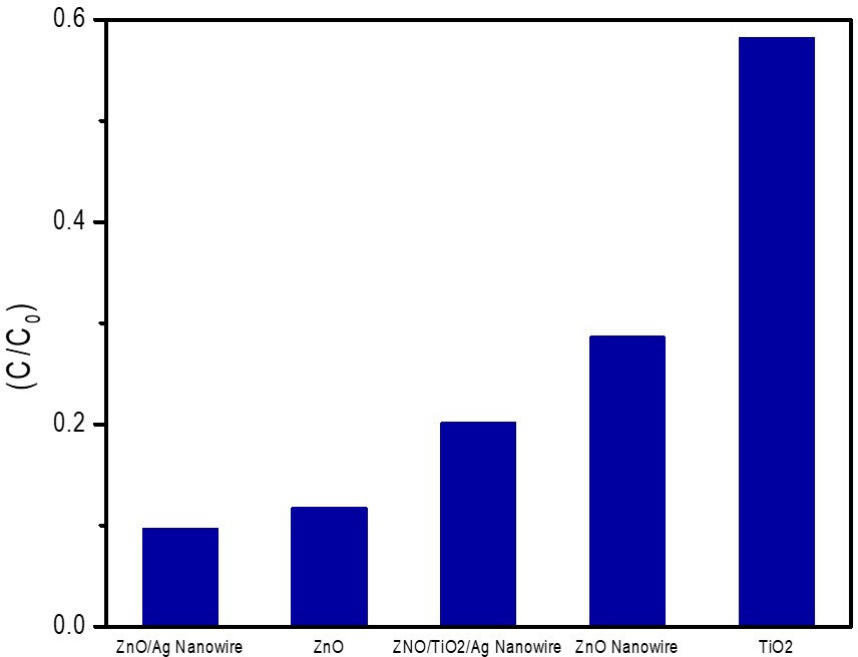
Comparative analysis of the photocatalytic efficiencies of different photocatalysts: ZnO, ZnO/TiO_2_/AgNW, ZnO nanowire, TiO_2_ and ZnO/AgNW composite.

To further substantiate the superior performance of the ZnO/AgNW composite, a comparative analysis was conducted against other well-known photocatalysts, including ZnO, ZnO/TiO_2_/AgNW, ZnO nanowire and TiO_2_, and the results are shown in figure 10. These results confirm the exceptional photocatalytic efficiency of the ZnO/AgNW composite, which achieved the lowest *C*/*C*_0_ value of 0.09707 after 40 min of UV irradiation. This value is significantly lower than those obtained with the other photocatalysts, clearly demonstrating the enhanced degradation capability of the ZnO/AgNW composite. This comparison highlights the synergistic effects of incorporating AgNWs into the ZnO matrix, which contribute to increased electron mobility, efficient charge separation and enhanced generation of ROS. These factors collectively enable more effective degradation of RhB under UV light, positioning the ZnO/AgNW composite as a highly potent photocatalyst, particularly in applications requiring efficient removal of organic pollutants from wastewater.

### 3.5. Mechanism of photocatalytic activity

The enhanced photocatalytic activity of the ZnO/AgNW composite can be attributed to several key mechanisms that are crucial for achieving efficient photocatalysis. Upon exposure to UV light, the incident photons excite the electrons in the VB of ZnO, causing them to jump to the CB and generate electron–hole pairs. The incorporation of AgNWs significantly enhances this process given its high SPR [38,39]. This SPR occurs when the conduction electrons in the AgNWs resonate with the incident light, generating strong localized electromagnetic fields. These fields enhance overall light absorption and generation of electron–hole pairs, thereby boosting the photocatalytic process.

**Figure 11.**
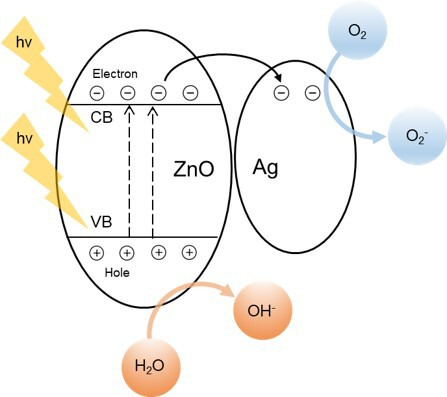
Schematic representation of the photocatalytic mechanism in the ZnO/AgNW composite under UV light.

The electrons generated in the CB of ZnO are efficiently transferred to the AgNWs, as depicted in figure 11 [40]. This process is facilitated by the formation of a Schottky junction at the ZnO–Ag interface, which creates an internal electric field [41–43]. This field drives the separation of the photogenerated charge carriers, ensuring that the electrons migrate towards the AgNWs while the holes remain within the ZnO matrix. The AgNWs then act as electron sinks and trap these electrons, preventing their recombination with the holes and prolonging their availability for the photocatalytic reactions. Once trapped by the AgNWs, the electrons interact with molecular oxygen (O_2_) adsorbed on the surface of the nanowire, resulting in the formation of superoxide anions (O_2_• ^−^). Simultaneously, the holes in the VB of ZnO react with water molecules (H_2_O) to produce hydroxyl radicals (•OH) [44,45]. These superoxide anions and hydroxyl radicals are both highly reactive species that play crucial roles in the decomposition of organic pollutants, such as RhB, into less harmful substances. The continuous generation and interactions of these ROS with pollutants facilitate efficient degradation, as illustrated in figure 11.

To further validate the superior performance of the ZnO/AgNW composite, it is useful to compare its photocatalytic activity with those of other well-known photocatalysts. For example, TiO_2_ is a widely studied photocatalyst that achieves a degradation efficiency of approximately 70% for RhB under UV light within 1 h of exposure. By contrast, the ZnO/AgNW composite achieves a significantly higher degradation efficiency of 90% under similar conditions. This enhancement is primarily attributed to the SPR effect of the AgNWs, which extends light absorption to the visible range and facilitates more efficient electron–hole pair generation than the wider bandgap of TiO_2_ [46].

ZnO has a bandgap of approximately 3.37 eV, which limits its absorption to the UV spectrum; however, the incorporation of AgNWs in the proposed composite narrows this bandgap slightly and introduces additional electron traps. These traps further reduce the electron–hole recombinations to boost the photocatalytic efficiency over that of pure ZnO. Although other materials, such as ZnO doped with Fe, have also shown improved photocatalytic activities, the ZnO/AgNW composite presented here demonstrates superior stability and reusability over multiple cycles, making it more practical for real-world applications [47]. These comparisons are supported by the mechanisms illustrated in figure 11 and underscore the enhanced photocatalytic performance of the ZnO/AgNW composite, thereby highlighting its potential in environmental remediation applications where efficiency, stability and reusability are critical [48].

## 4. Conclusions

This work reports the successful synthesis and characterization of ZnO/AgNW composites and demonstrates their efficacy in the photocatalytic degradation of RhB under UV irradiation. The composite film exhibits notably enhanced photocatalytic performance, which can be attributed to the synergistic effects of improved electron mobility, efficient charge separation and increased ROS generation facilitated by the integration of AgNWs with the ZnO matrix. These results contribute to the growing body of research on metal-enhanced semiconductor photocatalysts, reinforcing the idea that the incorporation of noble metals like Ag can significantly boost the photocatalytic efficiency by optimizing the charge-carrier dynamics and extending the active spectral range [49,50].

The demonstrated durability and reusability of the ZnO/AgNW composite further emphasize its potential as a practical and effective photocatalyst for environmental remediation. The consistent high performance across multiple cycles makes this composite particularly suitable for water treatment applications, where long-term stability and reliability are crucial. Therefore, future research related to this study could explore the photocatalytic activities of the proposed composite under varying environmental conditions and with different types of pollutants, paving the path for broader applications in environmental cleanup efforts [51].

## Data Availability

All data supporting this study, including raw data, processed data and analysis scripts, have been deposited in the Dryad repository [48].
